# Effects of Multimedia Audio and Video Integrated Orientation Training on Employees’ Organizational Identification and Self-Efficacy Promotion

**DOI:** 10.3389/fpsyg.2022.803330

**Published:** 2022-11-18

**Authors:** Ling-Chuan Huang, Chao-Yang Hung

**Affiliations:** ^1^College of Management, Da Yah University, Changhua, Taiwan; ^2^Department of International Business Management, Da Yah University, Changhua, Taiwan

**Keywords:** multimedia education, organizational commitment, education and training, satisfaction with education, regression analysis

## Abstract

This study investigates the instructional effectiveness of multimedia audio and video integrated pre-service education. In addition, the effects of pre-service education on organizational identification and self-efficacy promotion are discussed. The participants of the study consist of 264 supervisors and employees in the high-tech industry. The results revealed statistically significant positive effects of pre-service education on employees’ organizational identification and self-efficacy. The effect of employees’ organizational identification on self-efficacy promotion was also significantly positive.

## Introduction

The quality of human resources is key for an enterprise to gain an advantage in the competition (Ngoma and Dithan Ntale, 2016). As is the case in investment, enterprises should consider their benefit in education and training as well. The evaluation of the effectiveness of training programs is a primary issue. This is because, based on the results of the evaluation, enterprises can invest more resources to the enhancement of the quality of their workforce to take a step forward in the competition. In a society with a knowledge-based economy, learning is a source of power for the continuous progress of individuals, organizations, and society as well as a method of maintaining the advantage in competition (Onyishi et al., 2015). A company can improve the internal quality of their workforce and increase its competitiveness through various training programs. In addition to enhancing employees’ knowledge and skills at work and changing employees’ job attitude, education and training can be utilized for the future development of individuals and organizations (Koen et al., 2013). Education and training used to be used for work-related needs or urgently needed manpower. However, orientation training is now utilized to develop a more positive job attitude of employees for better work performance. It is broadly utilized in practice.

Traditional face-to-face courses are commonly used for the education and training of internal employees. Traditional teaching, being restricted to teaching materials, is relatively dull. Traditional training programs fall short in arousing learners’ interests. Modern enterprises should consider the application of multiple learning methods to satisfy employees’ learning needs and enhance the effect of learning with the help of multiple learning styles (Chen and Lim, 2012). Traditional learning styles with education and training were therefore impacted by the emergence of e-learning. They changed the nature of training programs in enterprises. With the popularity of the Internet, multimedia audio/video integrated learning styles are gradually replacing training in traditional classrooms. Many enterprises are starting to implement multimedia information technologies to respond to market changes rapidly and enhance their competitiveness. In order to increase employees’ learning efficiency, education and training has been transferred into multimedia audio/video environments. In addition to reducing labor costs for enterprises, employees’ learning is more flexible without the restrictions of time and place (Sylva et al., 2019).

Steven et al. (2016) indicated that organizations instructing their employees in professional knowledge through digital education and training can enhance professionalism and conformity while facilitating the willingness and integration of employees. Dechawatanapaisal (2018) also revealed that many enterprises tended to deliver professional knowledge to the employees through education and training to enhance corporate competitiveness and employees’ self-worth. Through these programs, they indirectly improve their employees’ sense of identity, cohesion, and connection with the company as well as enhancing employees’ self-efficacy and effectively improving their talents. For this reason, traditional orientation training might not satisfy employees’ needs. An enterprise should design a new orientation training model to make the employees be aware of themselves, be able to face challenges, and serve enthusiastically. In this way, they can further build their personal value and vision while hitting their targets. It is further discussed in this study that enhancing employees’ involvement in work, organizational identification, and satisfaction with self-efficacy promotion can be achieved through a newer model of training. Most of the previous research on the use of multimedia audio/video in education focused on the discussion of school education (Lohmann and Frederiksen, 2018; Kincey et al., 2019; Matsiola et al., 2019; Mei et al., 2019; Ocak and Baran, 2019) but rarely discussed education and training in enterprises. However, research indicated that employees participating in education and training can improve in terms of productivity (Becker, 1962; Smith and Kenyon, 2005; Zieba and Zieba, 2014), ability and knowledge (Mann and Robertson, 1996; Huang, 2001), and self-efficacy (Mann and Robertson, 1996). Nevertheless, previous literature scarcely mentioned improving the effectiveness of training styles nor the enhancement of employees’ job involvement, organizational commitment, and satisfaction with self-efficacy. The effect of multimedia audio and video integrated orientation training on employees’ organizational identification and self-efficacy promotion was investigated in this study. The results of the study are expected to help enterprises to practice orientation training effectively, enhance the internal operation efficiency, and successfully implement human resource development.

## Literature Review and Hypothesis

### Multimedia Audio/Video Teaching

Matsiola et al. (2019) defined multimedia audio/video teaching as teaching with materials commonly presented with texts (including printed text or dictated text) and images (containing illustrations, pictures, photos, maps, animation, or images). Multimedia audio/video learning referred to learning with texts and pictures. Multimedia audio/video learning was also called dual-code learning or dual-channel learning. In other words, multimedia audio/video presentation referred to messages being sent *via* texts and pictures. Multimedia audio/video teaching message or multimedia teaching presentations aid learning with the presentation of texts and pictures (Kincey et al., 2019). Mei et al. (2019) considered multimedia audio/video integrated teaching as computer multimedia audio/video or network technology. Such media technology had the advantages of digitalization, audio/visual sound and light multiple stimulation, easy access, fast processing, and convenience in communication. The implementation of integration meant the integration and application of teaching, i.e., being used as a teaching tool. Ocak and Baran (2019) explained that computer multimedia audio/video integrated teaching did not simply refer to teachers being able to use computers; it referred to teachers being able to more effectively achieve the instructional objectives with the help of computers. Lohmann and Frederiksen (2018) pointed out multimedia audio/video integrated teaching as the integration of information technologies into curricula, materials, and instruction in order to make the information technology become a vital teaching and learning tool for teachers and students. In addition, the use of information technology has become a part of daily teaching activity in classrooms.

### Multimedia Audio/Video Teaching Theory and Application

The information communication model was originally designed by Shannon and Weaver in 1949–1963 for industrial design. The application of one-way communication was then applied to teaching to explain the learning process (Hsu, 1999). Analyses revealed that, when explaining teaching processes with the Shannon-Weaver message model, the data of teachers, textbooks, speeches, music, or graphic images were the message sources, and each type of data could provide messages in various fields. However, different types of data sources could affect communication, i.e., the selection of teaching media (Hsu, 1999).

In the process of the communication model, it is clearly understood that the “source” is the first element in the entire communication process; in other words, the source of the communication message, which can be any persons or organizations. “Encoding” is the second element of communication. It is used in the process of changing the thought, data, or feeling, and is delivered from the source in the form of various symbols or behaviors understandable by senses. The message is the third element of the communication model. “Encoded” messages should be delivered through a selected “channel,” which is the fourth element. Various audiovisual media could be the “channel.” “Decoding” is the fifth element of the model. When a message is delivered from the channel to the destination, the recipient has to convert and reverse such symbols to correctly understand the message content. The “receiver,” as the sixth element of communication model, sends another message, after receiving the message. The purpose of this message is to ensure that the source understands the communication. It is the feedback mechanism.

Since people have different experiences due to their diverse educational and cultural backgrounds, effective communication can be generated in the “mutual experience” between source and receiver (Chang and Chu, 1998). In education, the theoretical research and practical application of communication should not be restricted to the surface message delivery of language or symbols. It should also contain the meaning of cultural message, machine message processing interface, human feeling, message processing in cognitive psychology, as well as the mutual effects between understanding the meaning and communication styles.

Teachers use different teaching media to find the clearest and most ideal communication channel with students. Students, on the other hand, can clearly understand the course content, and concept through the delivery of media material information. The selection of the most appropriate teaching media to achieve teaching goals is an important direction to follow. Relatedly, teachers need to understand the properties and characteristics of various teaching media in order to make the best of the auxiliary teaching materials and achieve the learning goals.

### Research on Orientation Training and Organizational Identification

Bi et al. (2019) explained orientation training as reinforcing employees’ knowledge, skills, and attitude through planned and systematic instruction. They added that it is the guidance to enhance work efficiency and ability in the application of knowledge to daily work. High awareness of organizational support can promote employees’ positive emotional integration of the organization and mean they devote their effort toward organizational profits. Sylva et al. (2019) regarded the creation of organizational performance through organizational identification. Employees who are aware of more profits shared in the organization can reveal organizational identification. The orientation training, as a way of intangible profit making, allowed the growth of employees beyond salaries. Employees who are aware of the gained profits can enable positive emotional connection with the organization and develop organizational identification. Georgiou and Nikolaou (2019) mentioned that the practice of orientation training can compensate for the gap between business performance and human resources of enterprises to a large extent. Additionally, it can improve employees’ lack of ability at work and enhance their professional knowledge. Furthermore, employees can explore their potential through orientation training to find the motivation for self-efficacy promotion and further facilitate the organizational identification. Accordingly, the following hypothesis is proposed in this study.

H1: Orientation training has significant positive effects on organizational identification.

### Research on Organizational Identification and Self-Efficacy

Fox et al. (2018) considered that employees with organizational identification can be highly motivated at work. They have a sense of pride and are willing to promote their self-efficacy to achieve the objectives at work. Kucherov and Manokhina (2017) stated that enterprises or organizations which are able to practice effective orientation training can enrich employees’ knowledge of management and professional technology, change their job attitude, and enhance organizational identification and loyalty. These, in turn, enhance the overall organizational performance. Meanwhile, the practice of employee education and training was the best way for an enterprise to improve the employees’ job competency, reinforce their production skills, and allow them to develop competency in the organizational system. As a result of this, the employees’ self-efficacy is promoted and they achieve the company-set objectives. Moke et al. (2018) indicated that training employees through effective orientation training can enrich employees’ management knowledge and professional technology, change their job attitude, and cultivate organizational identification and loyalty to the company. In addition, it enhances the overall performance of the organization to further develop the enterprise and acquire corporate competitiveness. Accordingly, the following hypothesis was proposed in this study.

H2: Organizational identification presents significantly positive effects on self-efficacy promotion.

### Research on Orientation Training and Self-Efficacy Enhancement

Byun and Ha (2019) mentioned that orientation training, based on the needs at work or for businesses, aimed to facilitate the individual skills or knowledge to achieve the preset requirements. They stated that it is a learning program for employees to learn work-related knowledge and technology. In addition, they mentioned that it is related to the ability development to enhance work performance, and they specifically emphasized the shaping of skills and methods. In the same vein, Al-Swidi and Yahya (2017) considered that orientation training aimed to improve employees’ working ability and to enable their immediate engagement in the new work environments which included adapting to new products, working programs, policies, and standards. In addition to the emphasis on performance, Peeters et al. (2019) regarded orientation training as the basis of teaching a certain model since employees naturally tend to perceive that training as standard. Training in the beginning when a newcomer is unaware can help employees understand the basic form and standard model. It emphasized the education and training of new employees. An enterprise providing more education and training can help the employees improve their motivation and self-efficacy to contribute to their understanding and promote their performance. According to the above-mentioned literature the following hypothesis was developed:

H3: Orientation training has significantly positive effects on self-efficacy promotion.

## Methodology

### Conceptual Architecture

**FIGURE 1:**
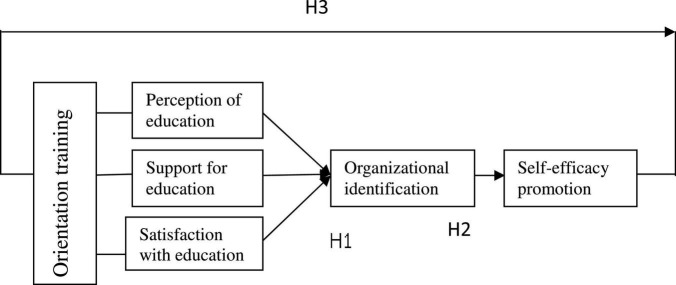
Conceptual structure.

Based on the literature on theory and application, the conceptual framework of this study was drawn (Figure 1).

### Operational Definition

#### Orientation Training

The education and training scale in this study refers to the one developed by Schmidt (2007). In this scale, the perception of training, organizational support for training, and satisfaction with training are included.

#### Organizational Identification

The organizational identification scale in this study refers to the definition and scale proposed by Mael and Ashforth (1992).

#### Self-Efficacy Promotion

The self-efficacy promotion scale in this study is the revised version of the scale proposed by Renkema et al. (2009). This scale contained the intention to participate in work-related and career-related development activities.

### Research Object

#### Sampled Object

The high-tech industry in Hsinchu Science Park was determined as the sample of this study. The industrial development in Hsinchu Science Park focuses on the integrated circuit, computer and peripherals, telecommunications, optoelectronics, precision machinery, and biotechnology. Since its establishment, the government has invested large amounts of funds in the software/hardware construction. It rapidly developed due to the convenient transportation and nice working environment as well as nearby academic and research institutions, such as National Yang Ming Chiao Tung University, National Tsing Hua University, and Industrial Technology Research Institute.

#### Questionnaire Retrieval

Supervisors and employees in high-tech industries were included in the research sample. The firms were determined with random sampling from industries in integrated circuit, computer and peripherals, telecommunications, optoelectronics, precision machinery, and biotechnology announced by Hsinchu Science Park. Among the enterprises in the industry, the ones applying multimedia audio/video integrated orientation training were randomly selected as the samples. They were distributed 50 copies of the questionnaire each. In total, 300 copies were distributed. The questionnaire was distributed and collected on site. A total of 264 valid copies were retrieved, with a retrieval rate of 88%.

### Analysis Method

Regression analysis was used in this study to understand the relations among orientation training, organizational identification, and self-efficacy enhancement. Through regression analysis, the effects of independent variables on dependent variables as well as independent variables on intervening variables and dependent variables were tested in this study. Regression analysis was utilized for the general understanding of the relationships among major variables of orientation training, organizational identification, and self-efficacy enhancement in this study. It was also used with a view to verify the existence of the effect.

## Results

### Reliability and Validity Analysis

According to Kaiser (1958), the evaluation standard for convergent validity should conform to four principles: (1) The eigenvalue of the extracted factor must be higher than 1; (2) the measured items in dimensions must be convergent to a common factor; (3) the factor loadings of the variables must be higher than 0.5; and 4. the cumulative variance explained must achieve 50%.

Gaski and Nevin (1985) indicated that the evaluation standards for discriminant validity should conform to two principles: (1) The correlation coefficient of the paired variables must be significantly smaller than 1, and (2) the correlation coefficient between any two dimensions must be smaller than Cronbach’s α of a single dimension.

#### Orientation Training

Cronbach’s α was used as a test of reliability in this study and was designed to measure orientation training. Through Cronbach’s α, the stability and consistency of items in the scale were tested. After the factor analysis, three factors were extracted. The first factor, “perception of education” (eigenvalue = 2.332, α = 0.95), had factor loadings of 0.958, 0.952, 0.950, and 0.771. The second factor, “support for education” (eigenvalue = 5.349, α = 0.94), had factor loadings of 0.954, 0.948, 0.947, and 0.754. The third factor, “satisfaction with education” (eigenvalue = 2.588, α = 0.94), had factor loadings of 0.964, 0.961, 0.955, and 0.717. The cumulative covariance explained by the three factors reached 85.578%. According to the results of the analyses, it can be stated that orientation training showed convergent validity in this study.

The correlation coefficients of the dimensions of orientation training were found to be between 0.242 and 0.314. The correlation coefficient between any two dimensions was smaller than Cronbach’s α of an individual dimension. The results indicated that there existed discriminant validity among dimensions.

#### Organizational Identification

Cronbach’s α was used to test the reliability of the organizational identification measurement instrument in this study. It was used to check the stability and consistency of the items in the scale. After factor analysis, one factor (eigenvalue = 4.970, α = 0.96) was extracted, with factor loadings of 0.935, 0.924, 0.906, 0.902, 0.898, and 0.894. The cumulative covariance explained reached 82.832%, which showed the convergent validity of organizational identification.

#### Increasing Self-Efficacy

The consistency of the items developed to measure the self-efficacy in the scale was tested using Cronbach’s α as a reliability test. After factor analysis, one factor was extracted (eigenvalue = 5.434, α = 0.98), and the factor loadings were found as 0.960, 0.956, 0.956, 0.949, 0.946, and 0.942. The cumulative covariance explained achieved 90.570%. The results revealed a convergent validity of self-efficacy.

### The Influence of Orientation Training on Organizational Identification

To test H1, VIF < 10 shows no collinearity. The analysis results in Table 1 revealed that the perception of education (Beta = 0.286^***^), support for education (Beta = 0.222^***^), and satisfaction with education (Beta = 0.162^**^) showed significant effects on organizational identification. Therefore, it can be concluded that H1 was supported.

**TABLE 1:** Analysis of the relationship between orientation training and organizational identification.

Dependent variable → Independent variable↓	Organizational identification	
Orientation training	Beta	P
Perception of education	0.286***	0.000
Support for education	0.222***	0.000
Satisfaction with education	0.162**	0.005
F	27.650	
Significance	0.000***	
*R* ^2^	0.242	
Adjusted *R*^2^	0.233	

**p < 0.01, ***p < 0.001.

*Self-organized in this study.*

### The Influence of Orientation Training and Organizational Identity on Self-Efficacy Promotion

#### Correlation Analysis of Orientation Training and Self-Efficacy Promotion

When testing H3, VIF < 10 showed no collinearity. The analysis results, which can be seen in Table 2, revealed significant effects of perception of education (Beta = 0.382^***^), support for education (Beta = 0.206^***^), and satisfaction with education (Beta = 0.171^**^) on self-efficacy promotion. Based on these results, it can be stated that H3 was supported.

**TABLE 2:** Analysis of orientation training, organizational identification, and self-efficacy promotion.

Dependent variable → Independent variable↓	Self-efficacy promotion			
Orientation training	Beta	*P*	Beta	*P*
Perception of education	0.382***	0.000		
Support for education	0.206***	0.000		
Satisfaction with education	0.171**	0.002		
Organizational identification			0.582***	0.000
*F*	41.312		134.079	
Significance	0.000***		0.000***	
*R* ^2^	0.323		0.339	
Adjusted *R*^2^	0.315		0.336	

**p < 0.01, ***p < 0.001.

*Self-organized in this study.*

#### Correlation Analysis of Organizational Identification and Self-Efficacy Promotion

When testing H2, VIF < 10 showed no collinearity. The analysis results in Table 2 revealed significant effects of organizational identification (Beta = 0.582^***^) on promoting self-efficacy. Therefore, H2 was supported.

## Discussion

Orientation training in high-tech industries can enhance employees’ organizational identification. High-tech businesses with orientation training can help their employees develop high organizational identification. Employees in high-tech industries receiving more frequent orientation training will have a deeper understanding of work execution to enhance their organizational identification. Employees in high-tech industries who identify themselves as members of the organization and agree with the organizational mission will regard the success of the organization as personal success. With stronger organizational identification, they can promote their self-efficacy more positively which, in turn, enhances personal performance, and enables them to achieve the organizational goals. Orientation training can enhance the willingness of employees’ self-efficacy promotion. Employees’ self-promotion can be the tool to increase the organizational value. The development in workplaces can facilitate organizational effectiveness and help the company to maintain a competitive advantage, which promotes the outputs of human resources (attitude, behavior, and human capital), organizational performance (performance and productivity), and financial performance (profit and financial indicators) (Maurer et al., 2003; Tharenou et al., 2007). For this reason, employees’ self-promotion provides significant contribution to organizational learning and knowledge-based competition (Tharenou et al., 2007). Employees positively engaging in continuous self-promotion to prevent their skills from falling short remain attractive to employers (Moos, 2009; Mustamin, 2012). It was also understood that understanding the incentives for self-promotion can help the organizations facilitate self-promotion. High-tech businesses with orientation training will have employees who show stronger willingness of self-efficacy promotion. High-tech businesses should regularly examine or improve the hardware facilities. Additionally, they should attempt to apply multimedia audio and video interactive teaching and materials or some relevant training facilities and teaching media. In this way, they can stimulate employees’ learning intention and interests in orientation training and achieve the objective of orientation training which improves the effectiveness of orientation training. For instance, the application of audio and video teaching is to deliver teaching content through electronic equipment of movies, video files, and recorders. High-tech businesses should emphasize employee orientation training as well as listing fixed percentages of expenses in the budget for purchasing facilities and equipment related to orientation training. By doing so, they can enhance the effectiveness of orientation training. Relevant research and the empirical results of this study discovered that there was more than one training method able to enhance the effectiveness of employee orientation training. Simultaneously using two or more training methods can effectively enhance employees’ satisfaction with orientation training (Zumrah et al., 2013; Cao and Hamori, 2016) and their technological ability (Hollenbeck et al., 2006) which promotes the application of their knowledge to work (Aarabi et al., 2013). However, there are certain points to be considered when establishing orientation training facilities and developing the relevant curricula. These include the form and content of orientation training, orientation training skills, selection of lecturers, and richness of hardware equipment.

## Theoretical Contributions of the Study

The research results revealed that organizational performance is created through organizational identification. Employees in high-tech industries who understand the profits shared in the organization can develop organizational identification. Employee orientation training in high-tech industries is worth a lot to employees; they engage more with their work and acquire extra benefits beyond the work in the orientation training. In orientation training, employees in high-tech industries psychologically feel like a member of the company. This feeling improves organizational identification. Orientation training in high-tech industries can boost the employees’ organizational identification and self-efficacy promotion. Employees who are highly aware of organizational support can develop positive emotional integration of the organization and devote effort toward organizational profits. In this case, the design of employee orientation training in high-tech industries can be reinforced to promote organizational identification and self-efficacy. When employees perceive the effects of their ideas on decision-making, they would engage more in the work to create maximal profits for the organization. In addition to the findings in this study, the employees’ orientation training can also enhance organizational identification, organizational reputation and characteristics (Smidts et al., 2001), organization-offered autonomy (Russo, 1998), organizational support (Wiesenfeld et al., 2001), employees’ psychological ownership (Johnson et al., 2006), seniority in the organization (Riketta, 2005), and individual-organization fit (Cable and DeRue, 2002) which can affect organizational identification. For this reason, managers should take them into consideration in practical application to promote organizational identification. According to the results, the following suggestions are proposed: high-tech industries should use education and training satisfaction surveys right after the end of orientation training. Through these surveys, they can understand the trained employees’ satisfaction with course materials, lecturers, supplementary materials, and teaching methods. Also, it can help emphasize the competency reinforcement and growth, review the difference between the target and the existing knowledge, and fill in this gap through training and development. Competency is not the same as expertise (knowledge, technique, capability). Another benefit of the study is in establishing an effective internal lecturer training program and system to improve the lecturers in terms of relevant training and teaching methods, and train lecturers’ communication skills. With the help of this program, the internal training and teaching quality in high-tech industries can be improved. It is also suggested that high-tech industries can promote competency-based and development-oriented performance evaluation systems. As a result of this, performance management and development can be recognized as an important reference for human capital development.

Previous research on multimedia audio- and video-integrated education focused on verifying the relationship between learning motivation and learning outcome, while the effects on organizational identification and self-efficacy promotion had not been verified. For this reason, this study brings in new elements to enrich existing theories. Most current studies on the relevant theories focus on school education but rarely on business organizations. Therefore, this study can contribute to the original theories. Moreover, this study, which targets multimedia audio and video integrated education, can verify employees’ organizational identification and self-efficacy promotion. The research results confirmed the theoretical statement that the original theories are supported by more empirical results. As for the practical contribution, the results revealed significant positive effects of organizational identification on increasing self-efficacy. It means that multimedia audio/video integrated orientation training can establish and reinforce employees’ organizational identification which facilitates employees’ self-efficacy. It also proves that the practice of multimedia audio/video integrated orientation training can boost employees’ organizational identification and self-efficacy in high-tech industries. In addition, it provides a measurement guideline for high-tech industries by reinforcing the employees’ organizational identification and self-efficacy enhancement.

## Data Availability Statement

The original contributions presented in the study are included in the article/supplementary material, further inquiries can be directed to the corresponding author/s.

## Ethics Statement

The present study was conducted in accordance with the recommendations of the ethics committee of the Da-Yah University, with written informed consent being obtained from all the participants. All the participants were asked to read and approved the ethical consent form before participating in the present study. The participants were also asked to follow the guidelines in the form in the research. The research protocol was approved by the ethical committee of the Da-Yah University.

## Author Contributions

L-CH performed the initial analyses and wrote the manuscript. C-YH assisted in the data collection and data analysis. Both authors revised and approved the submitted version of the manuscript.

## Conflict of Interest

The authors declare that the research was conducted in the absence of any commercial or financial relationships that could be construed as a potential conflict of interest.

## Publisher’s Note

All claims expressed in this article are solely those of the authors and do not necessarily represent those of their affiliated organizations, or those of the publisher, the editors and the reviewers. Any product that may be evaluated in this article, or claim that may be made by its manufacturer, is not guaranteed or endorsed by the publisher.

## References

[B1] Aarabi M. S. Subramamiam I. D. Akeel A. B. (2013). Relationship between motivational factors and job performance of employees in Malaysian service industry, Asian. *Soc. Sci.* 9 301–310. 10.5539/ass.v9n9p301

[B2] Al-Swidi A. Yahya M. A. (2017). Training transfer intention and training effectiveness. *Int. J. Organ. Anal.* 25 839–860. 10.1108/IJOA-07-2016-1043

[B3] Becker G. S. (1962). Investment in human capital: a theoretical analysis. *J. Polit. Econ.* 70 9–49. 10.1086/258724

[B4] Bi J. W. Liu Y. Fan Z. P. Zhang J. (2019). Conducting importance-performance analysis (IPA) through online reviews. *Tour. Manag.* 70 460–478. 10.1016/j.tourman.2018.09.010

[B5] Byun S. W. Ha Y. O. (2019). Factors influencing nurses’ intention to stay in general hospitals. *Korean J. Occup. Health Nurs.* 28 104–113.

[B6] Cable D. M. DeRue D. S. (2002). The convergent and discriminant validity of subjective fit perceptions. *J. Appl. Psychol.* 87 875–884. 10.1037/0021-9010.87.5.875 12395812

[B7] Cao J. Hamori M. (2016). The impact of management development practices on organizational commitment. *Hum. Resour. Manag.* 55 499–517. 10.1002/hrm.21731

[B8] Chang X. Chu Z. (1998). *Instructional Media.* Taipei: Wunan Book Publishing Company.

[B9] Chen D. J. Q. Lim V. K. G. (2012). Strength in adversity: the influence of psychological capital on job search. *J. Organ. Behav.* 33 811–839. 10.1002/job.1814

[B10] Dechawatanapaisal D. (2018). Examining the relationships between HR practices, organizational job embeddedness, job satisfaction, and quit intention: evidence from thai accountants. *Asia Pacific J. Bus. Adm.* 10 130–148. 10.1108/APJBA-11-2017-0114

[B11] Fox C. Webster B. D. Casper W. C. (2018). Spirituality, psychological capital and employee performance: an empirical examination. *J. Manag. Issues* 30 194–213.

[B12] Gaski J. F. Nevin J. R. (1985). The differential effects of exercised and unexercised power sources in a marketing channel. *J. Mark. Res.* 22 130–142. 10.2307/3151359

[B13] Georgiou K. Nikolaou I. (2019). The influence and development of psychological capital in the job search context. *Int. J. Educ. Vocat. Guid.* 19 391–409. 10.1007/s10775-018-9385-2

[B14] Hollenbeck G. P. McCall M. W. Jr. Silzer R. (2006). Leadership competency models. *Leadersh. Q.* 17 398–413. 10.1016/j.leaqua.2006.04.003

[B15] Hsu Z. (1999). *Instructional Media: System Design, Production and Application.* Taipei: Wunan.

[B16] Huang T. C. (2001). The relation of training practices and organizational performance in small and median size enterprises. *Educ. Train.* 43 437–444. 10.1108/00400910110411620

[B17] Johnson M. D. Morgeson F. P. Ilgen D. R. Meyer C. J. Lloyd J. W. (2006). Multiple professional identities: examining differences in identification across work-related targets. *J. Appl. Psychol.* 91 498–506. 10.1037/0021-9010.91.2.498 16551201

[B18] Kaiser H. F. (1958). The varimax criterion for analysis rotation in factor analysis. *Psychometrika* 23 187–200. 10.1007/BF02289233

[B19] Kincey S. D. Farmer E. D. Wiltsher C. Y. McKenzie D. Mbiza S. T. (2019). From chalkboard to digital media: the evolution of technology and its relationship to minority students’ learning experiences. *J. Faculty Dev.* 33 65–76.

[B20] Koen J. Klehe U. C. Van Vianen A. E. (2013). Employability among the long-term unemployed: a futile quest or worth the effort? *J. Vocat. Behav.* 82 37–48. 10.1016/j.jvb.2012.11.001

[B21] Kucherov D. Manokhina D. (2017). Evaluation of training programs in Russian manufacturing companies. *Eur. J. Train. Dev.* 41 119–143. 10.1108/EJTD-10-2015-0084

[B22] Lohmann S. Frederiksen L. (2018). Faculty awareness and perception of streaming video for teaching. *Collect. Manag.* 43 101–119. 10.1080/01462679.2017.1382411

[B23] Mael F. A. Ashforth B. E. (1992). Alumni and alma mater: a partial test of the reformulated model of organizational identification. *J. Organ. Behav.* 13 103–123. 10.1002/job.4030130202

[B24] Mann S. Robertson I. T. (1996). What should training evaluations evaluate? *J. Eur. ind. train.* 20 14–20. 10.1108/03090599610150264

[B25] Matsiola M. Spiliopoulos P. Kotsakis R. Nicolaou C. Podara A. (2019). Technology-enhanced learning in audiovisual education: the case of radio journalism course design. *Educ. Sci.* 9:62. 10.3390/educsci9010062

[B26] Maurer T. J. Weiss E. M. Barbeite F. G. (2003). Model of involvement in work-related learning and development activity: the effects of individual, situational, motivational, and age variables. *J. Appl. Psychol.* 88 707–724. 10.1037/0021-9010.88.4.707 12940410

[B27] Mei X. Y. Aas E. Medgard M. (2019). Teachers’ use of digital learning tool for teaching in higher education: exploring teaching practice and sharing culture. *J. Appl. Res. High. Educ.* 11 522–537. 10.1108/JARHE-10-2018-0202

[B28] Moke K. F. Chang K. W. Prihadi K. Goh C. L. (2018). Mediation effect of resilience on the relationship between self-efficacy and competitiveness among university students. *Int. J. Eval. Res. Educ.* 7 279–284. 10.11591/ijere.v7i4.15725

[B29] Moos L. (2009). “From successful school leadership towards distributed leadership,” in *School Leadership - International Perspectives*, ed. Huber S. (Dordrecht: Springer). 10.1007/978-90-481-3501-1_6

[B30] Mustamin Y. M. (2012). The competence of school principals: what kind of need competence for school success? *J. Educ. Learn.* 6 33–42. 10.1186/s13722-021-00225-x 33726843 PMC7968293

[B31] Ngoma M. Dithan Ntale P. (2016). Psychological capital, career identity and graduate employability in uganda: the mediating role of social capital. *Int. J. Train. Dev.* 20 124–139. 10.1111/ijtd.12073

[B32] Ocak C. Baran E. (2019). Observing the indicators of technological pedagogical content knowledge in science classrooms: video-based research. *J. Res. Technol. Educ.* 51 43–62. 10.1080/15391523.2018.1550627

[B33] Onyishi I. E. Enwereuzor I. K. Ituma A. N. Omenma J. T. (2015). The mediating role of perceived employability in the relationship between core self-evaluations and job search behaviour. *Career Dev. Int.* 20 604–626. 10.1108/CDI-09-2014-0130

[B34] Peeters E. Nelissen J. De Cuyper N. Forrier A. Verbruggen M. De Witte H. (2019). Employability capital: a conceptual framework tested through expert analysis. *J. Career Dev.* 46 79–93. 10.1177/0894845317731865

[B35] Renkema A. Schaap H. Dellen T. V. (2009). Development intention of support staff in an academic organization in the Netherlands. *Career Dev. Int.* 14 69–86. 10.1108/13620430910933583

[B36] Riketta M. (2005). Organizational identification: a meta-analysis. *J. Vocat. Behav.* 66 358–384. 10.1016/j.jvb.2004.05.005

[B37] Russo T. C. (1998). Organizational and professional identification: a case of newspaper journalists. *Manag. Commun. Q.* 12 72–111. 10.1177/0893318998121003

[B38] Schmidt S. W. (2007). The relationship between satisfaction with workplace training and overall job satisfaction. *Hum. Resour. Dev. Q.* 18 481–498. 10.1002/hrdq.1216

[B39] Smidts A. Pruyn A. T. H. Van Riel C. B. (2001). The impact of employee communication and perceived external prestige on organizational identification. *Acad. Manage. J.* 44 1051–1062. 10.2307/3069448

[B40] Smith E. Kenyon R. (2005). The business benefits of apprenticeships: the English employers’ perspective. *Educ. Train.* 47 366–373. 10.1108/00400910510601931

[B41] Steven D. C. Russell P. G. Ryan D. Z. (2016). Plugged in ordisconnected? A model of the effects of technological factors on employee job embeddedness. *Hum. Resour. Manage.* 55 109–126. 10.1002/hrm.21716

[B42] Sylva H. Mol S. T. Den Hartog D. N. Dorenbosch L. (2019). Person-job fit and proactive career behaviour: a dynamic approach. *Eur. J. Work. Organ. Psychol.* 28 631–645. 10.1080/1359432X.2019.1580309

[B43] Tharenou P. Saks A. Moore C. (2007). A review and critique of research on training and organizational level outcomes. *Hum. Resour. Dev. Rev.* 17 251–273. 10.1016/j.hrmr.2007.07.004

[B44] Wiesenfeld B. M. Raghuram S. Garud R. (2001). Organizational identification among virtual workers: the role of need for affiliation and perceived work-based social support. *J. Manage.* 27 213–229. 10.1177/014920630102700205

[B45] Zieba K. Zieba M. (2014). Training services in small and medium-sized enterprises: evidence from Poland. *Soc. Sci.* 84 47–56. 10.5755/j01.ss.84.2.7492

[B46] Zumrah A. R. Boyle S. Fein E. C. (2013). The consequences of transfer of training for service quality and job satisfaction: an empirical study in the malaysianss public sector. *Int. J. Train. Dev.* 17 279–294. 10.1111/ijtd.12017

